# Preclinical and clinical studies of a tumor targeting IL-12 immunocytokine

**DOI:** 10.3389/fonc.2023.1321318

**Published:** 2024-01-08

**Authors:** Christine M. Minnar, Grace Lui, James L. Gulley, Jeffrey Schlom, Sofia R. Gameiro

**Affiliations:** Center for Immuno-Oncology, Center for Cancer Research, National Cancer Institute, National Institutes of Health, Bethesda, MD, United States

**Keywords:** IL-12, NHS-IL12, M9241, PDS0301, cancer immunotherapy, cytokine, necrosis

## Abstract

The clinical success of immune checkpoint inhibitors (ICIs) has demonstrated the promise and challenges of cancer immunotherapy. There is an unmet need to develop novel cancer therapies that can provide clinical benefit for most patients with solid malignancies, which harbor innate or acquired resistance to ICIs. Interleukin-12 (IL-12) is a promising cytokine for cancer therapy given its direct stimulatory effects on innate and adaptive immunity. However, unfavorable pharmacokinetics and a narrow therapeutic index render recombinant IL-12 (rIL-12) less attractive as a cancer therapy. NHS-IL12 is a fusion protein of IL-12 and NHS76 (human IgG1) antibody engineered to target single and double stranded DNA present in necrotic areas solid tumors. In preclinical tumor models, NHS-IL12 elicited significant Th1 immune activation and tumor suppressive effects, primarily mediated by NK and CD8^+^ T lymphocytes, with engagement of myeloid immunity. NHS-IL12 is currently being evaluated clinically in combination with various therapeutic modalities, including chemotherapy, radiation therapy, immune checkpoint inhibition, vaccines, and epigenetic modulation. Here we review the preclinical and clinical studies involving NHS-IL12 for the treatment of solid malignancies.

## 1 Introduction

Inhibition of immune checkpoints has demonstrated unprecedented clinical benefit for a minority of cancer patients across malignancies, underscoring the promise and challenges of cancer immunotherapy. Most patients with solid cancers are refractory to immune checkpoint inhibitors (ICIs) or develop resistance after an initial clinical response. Thus, there is an unmet clinical need for novel therapies able to overcome mechanisms of tumor immune evasion and ICI resistance such as poor lymphocyte homing and function, defective tumor interferon gamma (IFNγ) signaling and MHC class I expression, and dysregulation in antigen processing and presentation. The use of novel combination therapy strategies involving tumor-targeted delivery of pro-inflammatory cytokines may dampen these barriers and attain clinical benefit for the treatment of solid malignancies.

**Figure 1:**
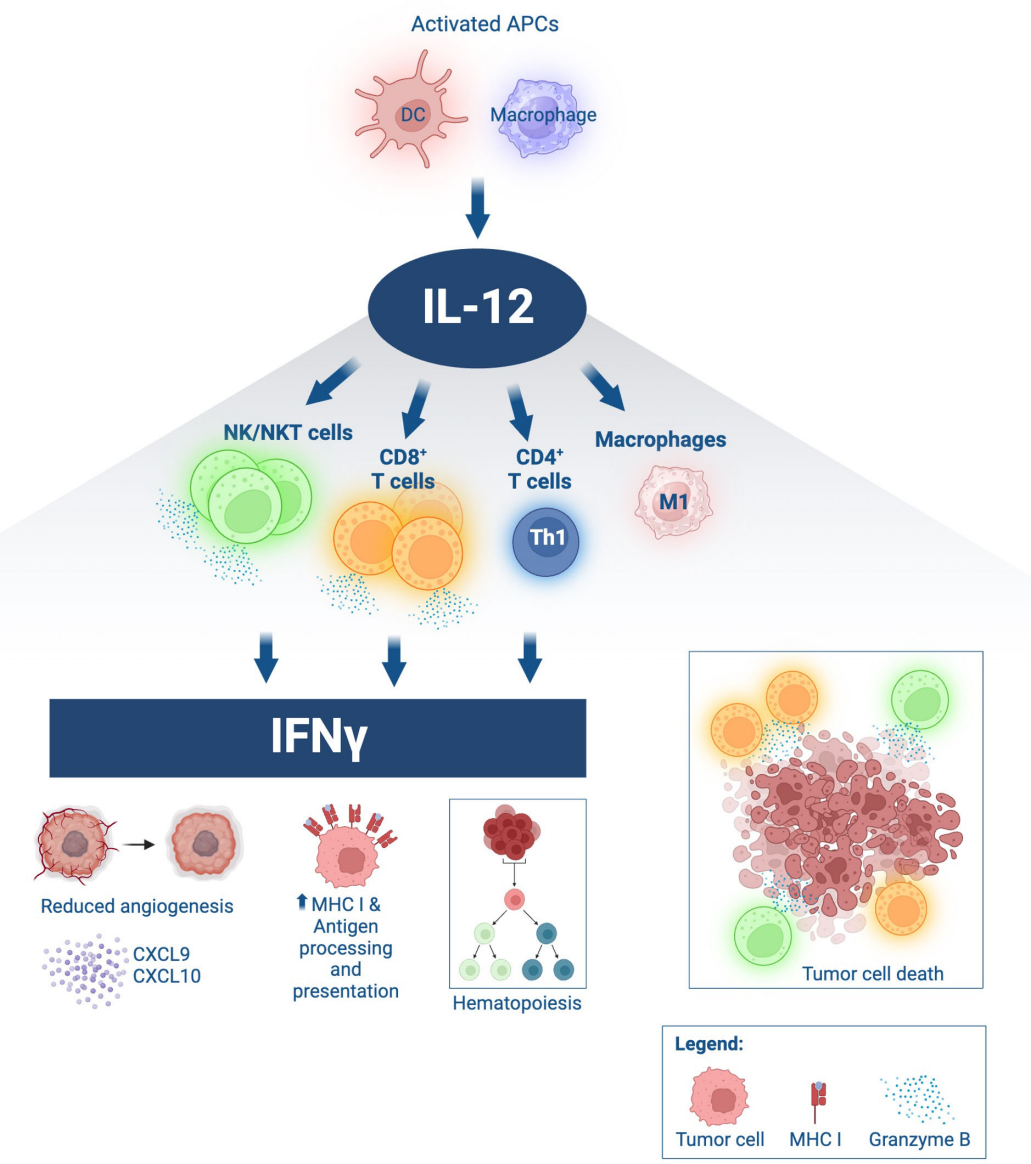
Immune effects of IL-12. IL-12 is produced by activated antigen-presenting cells (APCs), such as dendritic cells (DC) and macrophages. Upon binding to IL-12 receptors on lymphocytes, IL-12 induces proliferation, activation, and effector functions, including heightened cytolytic ability of NK and CD8^+^ T cells *via* granzyme B secretion leading to tumor cell death. IL-12 promotes Th1 differentiation in CD4^+^ T cells. IL-12 drives macrophage polarization towards an antitumor M1 phenotype. IL-12 signaling in lymphocytes induces significant IFNγ production, with downstream effects including decreased angiogenesis, secretion of lymphocyte-attracting chemokines such as CXCL9 and CXCL10 by activated myeloid cells, hematopoietic modulation, increased expression of MHC class I in target cells and improved antigen processing and presentation. Schematic produced in BioRender.com.

Interleukin-12 (IL-12) is a promising cytokine for cancer therapy. It is a member of the common receptor gamma chain family together with IL-2, IL-4, IL-7, IL-9, and IL-21. This family of cytokines elicits a broad spectrum of activity in both innate and adaptive immunity, with important clinical implications, reviewed elsewhere (1, 2). First isolated in 1989 from a B cell line with potent natural killer (NK) cell stimulatory properties (3), IL-12 is a cytokine consisting of p35 and p40 subunits linked by disulfide bridges forming the IL-12p70 heterodimer (4). IL-12 signals through the low affinity IL12Rβ1 and the high affinity IL12Rβ2, expressed on activated T and NK cells. Over the last three decades, IL-12 has gained widespread recognition for its pleiotropic effects able to simultaneously engage innate and adaptive immunity (**Figure 1**). IL-12 can induce T helper cell type 1 (Th1) pro-inflammatory immune responses that have been demonstrated to be beneficial for the treatment of infectious diseases, autoimmune disorders, and cancer. Produced by activated antigen presenting cells (APCs), such as dendritic cells (DCs), macrophages, B cells and neutrophils, IL-12 can potently activate T and NK cells, thereby increasing their proliferation and effector functions and inducing the release of IFNγ. The production of high levels of IFNγ can further orchestrate an antitumor milieu and create a feedback loop between IL-12 and IFNγ, increasing expression of major histocompatibility class I and II molecules (MHC-I/MHC-II) and tumor antigen presentation and recognition. Additionally, IL-12 has been shown to impede and reprogram immunosuppressive tumor associated macrophages (TAM) and myeloid-derived suppressor cells (MDSCs). IFNγ-stimulated macrophages can further release CXCL9 and CXCL10 chemokines that are anti-angiogenic and promote lymphocyte chemotaxis and differentiation (5–7).

Here, we review the current knowledge on the use of IL-12 as a cancer therapy, with a particular focus on the mechanism(s) of action and preclinical and clinical development of tumor-targeted NHS-IL12 (recently designated PDS0301, formerly known as M9241).

## 2 Preclinical studies with IL-12 therapy

### 2.1 Recombinant IL-12 as a cancer therapy

While preclinical investigations demonstrated robust immunostimulatory effects of human recombinant IL-12 (rhIL-12) in hematologic and solid malignancies (8–12), early clinical trials in cancer patients revealed that systemic delivery of rhIL-12 as a single agent resulted in severe toxicity and dampened response rates correlating to dose-dependent toxic levels of IFNγ and the immunosuppressive cytokine IL-10, respectively (13–16). Additionally, neither intravenous (i.v.) nor subcutaneous (s.c.) administration of rhIL-12 at the maximum tolerated dose (MTD) was able to achieve immunomodulatory changes within the tumor microenvironment (TME) (17).

### 2.2 Tumor-targeted IL-12 therapeutics

Various engineered IL-12 agents and delivery strategies, including monovalent IL-12 immunoglobulin Fc fusion protein with extended half-life, as well as chimeric antigen receptor (CAR-T) cells have been developed to better utilize the potent biological effects of endogenous IL-12 while limiting associated systemic toxicity by directing IL-12 to the TME (18–29). Benefits of tumor-targeted IL-12 include expansion of a local immune response, dampening systemic toxicity, thwarting local immunosuppressive mechanisms, and directly engaging T cells at the site of the tumor (16). As reviewed below, NHS-IL12 is among the most clinically advanced tumor-targeted immunocytokine therapies, with multiple ongoing clinical trials involving patients across various cancer types.

## 3 Preclinical studies involving NHS-IL12

### 3.1 NHS-IL12 as single agent

**Figure 2:**
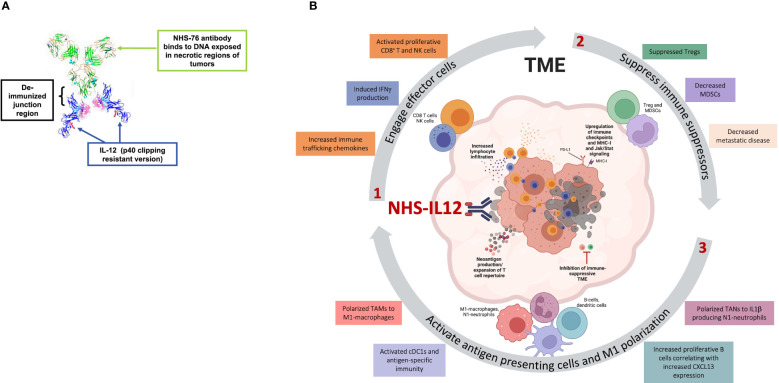
Effects of NHS-IL12 in the tumor microenvironment (TME). **(A)** Ribbon diagram of NHS-IL12, an immunocytokine composed of two IL-12p70 heterodimers fused to NHS76, an antibody targeting exposed DNA in tumor necrotic regions. **(B)** Proposed mode of action of NHS-IL12. 1. Necrosis-targeted IL-12 engages effector cells such as CD8^+^ T and natural killer (NK) cells within the TME. Upon activation, these immune subsets display increased proliferative and cytolytic capability and high IFN*γ* production. 2. IL-12 impedes immune suppressors such as myeloid-derived suppressor cells (MDSCs) and regulatory T cells (Tregs), thereby dampening metastatic disease. 3. IFNγ production by effector cells further orchestrates the activation of antigen presenting cells (APCs). Tumor-associated macrophages (TAMs) and neutrophils (TANs) polarize towards antitumor phenotypes such as M1-macrophages or N1-neutrophils. IFNγ activates conventional type 1 dendritic cells (cDC1) and their antigen-specific immunity and can increase proliferative B cells. Moreover, activated APCs produce chemokines such as CXCL9, CXCL10 and CXCL13, which further recruit effector cells into the TME and secrete endogenous IL-12, thereby promoting an IL-12/IFNγ pro-inflammatory feedback loop supportive of tumor suppression. Panel A was adapted from Greiner et al. Immunotargets Ther, 2021. Copyright ^©^ 2021, Dove Medical Press Limited. Schematic in panel B was assembled in PowerPoint and produced partially in BioRender.com.

NHS-IL12 is an immunocytokine encompassing two human IL-12 heterodimers linked at the C terminus to NHS76 (**Figure 2A**), an antibody targeting exposed DNA-histone complexes present in necrotic areas of solid tumors, thereby directing IL-12 to the TME (17, 30–32). NHS76 is a human IgG1 monoclonal antibody engineered with similar binding characteristics to chimeric TNT-1, a humanized antibody demonstrated to clinically target lung lesions upon intravenous administration (31–34). Fusion of IL-12 to NHS76 using a short linker aimed to reduce IL-12 bioactivity with resulting lower IFNγ production and its associated hallmark toxicities. Engineering of IL-12 into an immunocytokine further aimed at increasing its half-life relative to rIL-12 (17, 35). The absence of human IL-12 cross-reactivity to murine IL-12 receptors led to the development of an NHS-IL12 surrogate for preclinical studies, with NHS76 fused with murine IL-12.

Preclinical studies have demonstrated NHS-IL12 to elicit a spectrum of immune effects conducive to tumor suppression (**Figure 2B**). In its first demonstration of targeted delivery of IL-12, NHS-IL12 was shown to induce superior antitumor activity in LLC (Lewis lung carcinoma), MC38 (colon carcinoma) and B16 (melanoma) tumor models versus rIL-12, including after a single dose (35). Antitumor effects were dose-dependent and associated with increased maturation of splenic DCs, splenic and tumor-infiltrated NK cells, and CD8^+^ T cells. Responses in MC38 and B16 tumors showed a p15E-antigen specific CD8^+^ T-cell response with acquired memory during rechallenge (35). Additional studies in an orthotopic MB49 bladder cancer model showcased significant tumor regression with NHS-IL12 associated with decreases in immunosuppressive tumor TGFβ levels, MDSCs and TAMs, while increasing M1-macrophages and CD4^+^ and CD8^+^ T cell responses (17, 36). Overall, NHS-IL12-mediated tumor suppression in murine hosts is intrinsically related to IFNγ levels in a dose and tumor model-dependent manner. High IFNγ levels can manifest NHS-IL12 toxicity, as determined by transient mild to moderate decrease in body weight and hunch posture.

In a dose-escalation study of dogs with melanoma, s.c. NHS-IL12 (0.4, 0.8, 1.6, and 2.4 mg/m^2^) demonstrated antitumor activity and safe applicability, with a defined tolerable dose of 0.8 mg/m^2^ (37). A dose-dependent association between serum IFNγ levels, NHS-IL12 exposure, and presence of adverse events (including alteration in liver enzymes, fever, vasculitis and thrombocytopenia was observed. Studies in humanized mice with rhabdomyosarcoma further demonstrated tumor control *via* cancer cell senescence and greater survival dependent on Th1 responses (38, 39). *In vitro* studies stimulating human peripheral blood mononuclear cells (PBMCs) with NHS-IL12 showed decreased production of IFNγ compared to equimolar concentrations of rIL-12 inferring lower associated toxicities (35). Treatment of non-human primates with either s.c. or i.v. NHS-IL12 administration demonstrated safe and sustained pharmacokinetics, with increased half-life versus rIL-12 (17, 35, 36). NHS-IL12 was well tolerated, with a minor decrease in platelet counts observed, absence of fever and no significant injection site reactions (35). Together, these initial studies provided the rationale for the clinical development of NHS-IL12 and for the investigation of combinational approaches with NHS-IL12, such as radiation therapy, chemotherapy, vaccine, other immunotherapies, and epigenetic modifiers to build upon these initial findings.

### 3.2 NHS-IL12 in combination with standard-of-care therapies

Preclinical studies have examined the rationale for NHS-IL12 combination with tumor necrosis-inducing experimental and standard of care (SOC) therapies aimed at increasing IL-12 targeting to the TME. Radiation therapy was shown to increase necrosis and localization of NHS-IL12 in A204 (rhabdomyosarcoma) xenograft tumors and seen with synergistic antitumor activity in both A204 and LLC tumor models (35, 40). While more thorough investigations are still needed, published preliminary findings showed NHS-IL12 to have synergistic antitumor efficacy with the chemotherapeutic agents docetaxel, sunitinib and gemcitabine in MC38, Renca (renal cell carcinoma) and Panc02 (pancreatic adenocarcinoma) tumor models, respectively (35). These findings support clinical translation of NHS-IL12 combination with first-in-line therapies.

### 3.3 NHS-IL12 in combination with other immune oncology agents

The programmed cell death 1 (PD-1) and programmed cell death ligand 1 (PD-L1) proteins are IFNγ-inducible immune checkpoints that inhibit NK and T cell responses. Checkpoint blockade therapies targeting the PD-1/PD-L1 axis can dampen these immunosuppressive mechanisms and achieve antitumor efficacy in a minority of cancer patients with solid tumors (41, 42). A challenge for patients with resistance to these therapies is the low or absent lymphocyte infiltration level within the tumor. For this reason, NHS-IL12 in combination with the PD-L1-targeted monoclonal antibody avelumab was investigated as a strategy to enhance tumor-directed immunity and antitumor activity elicited by checkpoint inhibition. This combination elicited synergistic antitumor efficacy associated with increased immune infiltration and expansion of Th1 cytokines, tumor-specific immune memory, CD8^+^ T and NK cell proliferation, and enhancement of tumor antigen-specific CD8^+^ T cells (43, 44). Subsequent studies examined the combination of NHS-IL12 with bintrafusp alfa in multiple preclinical tumor models (45, 46). Bintrafusp alfa is a first-in-class bifunctional fusion protein of monoclonal human IgG1 antibody blocking PD-L1 fused to TGFβRII extracellular domains, thereby sequestering the immunosuppressive cytokine, TGFβ (45, 47). The combination of NHS-IL12 and bintrafusp alfa elicited significant tumor homing of effector memory CD8^+^ T cells, tumor-specific immunity, increased survival, and anti-metastatic effects and development of protective immune memory (45, 46). The addition of the vaccine PDS0101 targeting HPV16 E6/E7 to the combination of NHS-IL12 plus bintrafusp alfa was investigated in two human papillomavirus E6 and E7 positive (HPV E6/E7^+^) tumor models, TC-1 (lung carcinoma) and mEER (oropharyngeal squamous cell carcinoma). Triple therapy synergized for maximal antitumor efficacy and greater increases in tumor T cell infiltration. This report also confirmed previous findings that NHS-IL12 alone can greatly increase T-cell repertoire. The addition of a vaccine component further enhanced T cell clonality and expansion of antigen-specific T cells (48). In the non-HPV E6/E7^+^ tumors, EMT6 and MC38, the combination of NHS-IL12 and bintrafusp alfa synergized with antitumor efficacy and increased tumor-infiltrating CD8^+^ T cells. Increases in T and NK cells were observed peripherally as well. This combination was also shown to decrease metastatic disease in 4T1 tumors (46). Overall, the combination of NHS-IL12 with other therapeutics such as ICIs and vaccine therapies, induces synergistic antitumor effects including immune stimulation and infiltration into tumors.

### 3.4 NHS-IL12 and epigenetic modifiers

**Figure 3:**
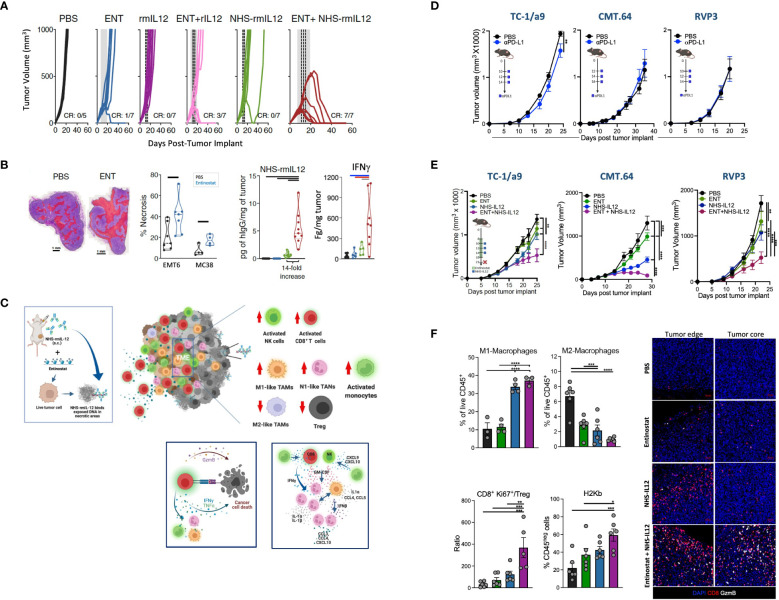
Tumor suppression elicited by NHS-IL12 in combination with entinostat in preclinical models. **(A)** Individual tumor growth curves of orthotopic EMT6-tumor-bearing mice treated with NHS-rmIL12 and/or entinostat (ENT). Gray shaded area indicates ENT treatment and dashed line depicts NHS-rmIL12 or recombinant murine (rm) IL-12 dosing. CR, complete response. **(B)** *(Left to right)* Representative hematoxylin and eosin (H&E) images of EMT6 tumors treated with PBS or ENT with areas of necrosis indicated in red. Necrosis quantification in individual EMT6 and MC38 tumors after 14 days on ENT. Graph shows quantification of human IgG in the tumor microenvironment (TME) from individual EMT6 tumor-bearing mice 2 days after NHS-IL12 treatment end. Right graph depicts IFNγ levels in the TME supernatant 6 days after last NHS-IL12 treatment. Graph bars denote *P* < 0.05. C. Proposed mechanism of action of NHS-IL12 in combination with entinostat. Entinostat-induced necrosis enables increased deposition of NHS-IL12 at the tumor site, leading to increased IFNγ in the TME. Combination therapy promotes significant activation of NK and CD8+ T cells infiltrating the TME, with engagement and polarization of multiple myeloid cell types at the tumor site towards an IFNγ antitumor biology, such as monocytes, M1 macrophages, and N1 neutrophils. Increased production of multiple pro-inflammatory cytokines and chemokines in the TME allows for a dynamic crosstalk between lymphocytes and myeloid cell populations, collectively synergizing to promote tumor suppression and eradication. **(D)** Antitumor effects of αPD-L1 therapy against TC-1/a9 (HPV16+), CMT.64 (lung), and RVP3 (sarcoma) tumors harboring multiple defects in MHC I expression, antigen processing and presentation, and IFNγ signaling. Graphs depict tumor growth mean ± S.E.M. Stars denote *P* < 0.05 (2-way ANOVA). **(E)** Antitumor effects of combination therapy against TC-1/a9, CMT.64, and RVP3 tumors. Graphs depict mean ± S.E.M. Stars denote *P* < 0.05 (2-way ANOVA). **(F)** Frequency of M1 and M2 macrophages, ratio of proliferative CD8^+^ T cells/CD4^+^ regulatory T cells (Treg), and MHC I expression in tumor cells from individual mice harboring TC-1/a9 tumors treated with NHS-IL12 and/or entinostat. Immunofluorescence image shows infiltration of CD8^+^ T cells and associated granzyme B expression in TC-1/a9 tumors treated with NHS-IL12 and/or entinostat. Graphs depict mean ± S.E.M. Stars denote *P<0.05, **P<0.01, ***P<0.001 (1-way ANOVA). **(A, B)** were adapted from Hicks et al., Nat Commun, 2021. Copyright ^©^ 2021. This is a U.S. Government work and not under copyright protection in the US. https://creativecommons.org/licenses/by/4.0/. **(C)** was produced in BioRender.com. **(D–F)** were adapted from Minnar et al., J ImmunoTher Cancer, 2022. Copyright ^©^ 2022, BMJ Publishing Group Ltd & Society for Immunotherapy of Cancer. All rights reserved.

Two recent reports examined the combination of NHS-IL12 with entinostat, a class I histone deacetylase (HDAC) inhibitor. This epigenetic modifier was chosen not only for its ability to facilitate necrosis but also for its capacity to inhibit immunosuppressive regulatory T cells (Tregs) and MDSCs and upregulate tumor cell MHC-I and antigen processing machinery (APM) (49–51). The combination of entinostat and NHS-IL-12 was demonstrated to induce robust suppression of tumors with a range of immunogenicity and sensitivity to PD-L1 blockade, including EMT6 (breast), CT26 (Kras G12D) and MC38 (colon), also inducing tumor-specific protective memory (52). Notably, whereas this combination therapy led to full resolution of large orthotopic “cold” EMT6 breast tumors in all mice, this effect was not seen with the combination of rmIL-12 with entinostat, underscoring the requirement for tumor targeting of IL-12 for effective tumor suppression (**Figure 3A**). Combination therapy with the tumor-targeted immunocytokine showed sustained NHS-IL12 localization in tumors compared to NHS-IL-12 monotherapy, which resulted in a sustained proinflammatory milieu with elevated IFNγ (**Figure 3B**). These effects were associated with increased tumor-infiltrating CD8^+^ T cells (TILs) and decreased Tregs. These cytotoxic and proliferative CD8^+^ TILs displayed increased and sustained functionality relative to those from NHS-IL12 monotherapy-treated tumors. Most importantly, single cell transcriptomic and proteomic analysis of EMT6 tumors demonstrated an increase in chemokines driving T-cell trafficking contributed by monocytes, macrophages, and neutrophils, including CCL5, CXCL9, and CXCL10. Macrophages were shown to be overwhelmingly polarized towards an M1 phenotype and gene program, requiring CD8^+^ T cells for this polarization (52). Combination therapy was shown to efficiently promote engagement of multiple myeloid cell populations in the TME, with significant activation of monocytes, M1 macrophages, and N1 neutrophils, collectively contributing to an interferon response involving IFNγ, IFNα, and IFNβ. Collectively, these observations indicated this combination therapy to induce a dynamic crosstalk initiated by activated lymphocytes to engage and modulate multiple myeloid cell populations from an immunosuppressive program into a cooperative and effective antitumor synergy (**Figure 3C**).

Due to the sustained increases of IFNγ noted with the combination of entinostat and NHS-IL12, a follow-up study investigated this combination therapy in TC-1/a9 (HPV E6/E7^+^), CMT.64 and RVP3 tumor models resistant to PD-1/PD-L1 blockade due to varying MHC-I and APM deficiencies (53) (**Figures 3D, E**). While many of the findings here mirrored previous findings of the important role of tumor-infiltrating CD8^+^ T cells, M1 macrophages, and T cell trafficking chemokines, this investigation demonstrated that the sustained pro-inflammatory response of entinostat and NHS-IL12 may be utilized for patients harboring innate and acquired resistance to checkpoint blockade therapies targeting the PD-1/PD-L1 axis. Analysis of the tumor immunome demonstrated combination therapy to efficiently polarize TAMs towards an M1 phenotype while dampening infiltration of M2 TAMs. Tumors from mice treated with combination therapy displayed significant infiltration of activated CD8+ T cells, resulting in a marked increase in CD8/Treg ratio in the TME. Notably, while NHS-IL12 therapy enabled significant increases in the number of CD8^+^ TILs at the tumor margin, combination therapy facilitated the infiltration of CD8^+^ TILs in the tumor core, now expressing granzyme B. These observations support increased cytolytic ability by CD8^+^ lymphocytes in the TME, now able to eliminate tumor cells given their partial recovery of the MHC I allele H-2Kb (**Figure 3F**). Additionally, this study demonstrated the role of NK cells and various antigen presenting cell populations, such as dendritic and B cells. Bulk RNAseq analysis of TC-1/a9 tumors showed enrichment of antigen processing and presentation, in addition to IFNγ and Jak/Stat signaling pathways, often dysregulated in checkpoint blockade resistance (53, 54). Together, these studies demonstrated the translatability of this combination for the treatment of patients harboring solid malignancies, including those resistant to PD-1/PD-L1-targeted therapies.

Overall, preclinical studies have demonstrated NHS-IL12 to accumulate in the TME, which can be facilitated by combination with necrosis-inducing therapies. NHS-IL12 activates CD8^+^ T and NK cells, promotes immune trafficking and pro-inflammatory cytokines including IFNγ, and CXCL9, CXCL10 and CXCL13 chemokines. In addition, NHS-IL12 supports polarization of TAMs and MDSCs towards a tumor-suppressive phenotype and increases antigen presentation for augmented tumor suppression across preclinical tumor models.

## 4 Clinical studies involving NHS-IL12

### 4.1 Phase I clinical studies

**Figure 4:**
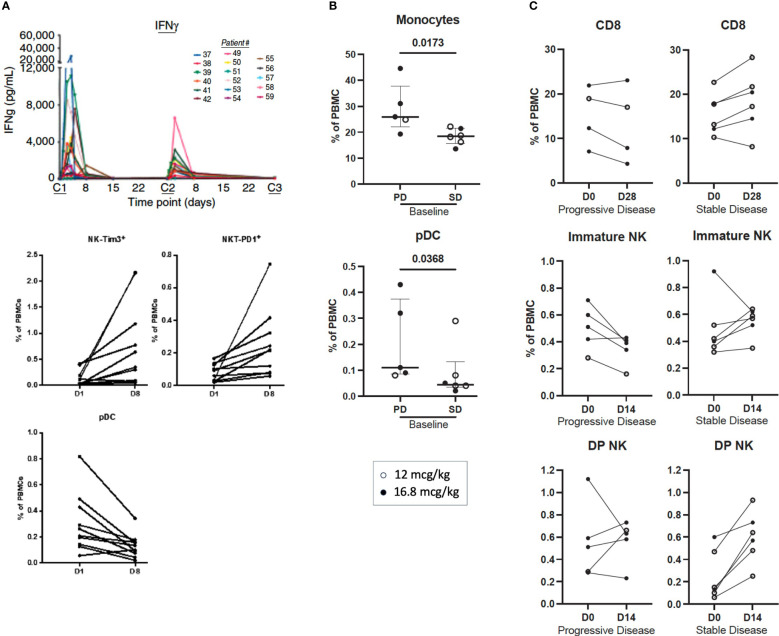
Immune correlates of clinical activity in advanced solid cancer patients treated with subcutaneous NHS-IL12 in the first-in-human clinical study. **(A)** (*upper panel*) Time-dependent rise in serum IFNγ levels during the first 2 cycles of NHS-IL12 therapy in 17 patients treated at the maximum tolerated dose (MTD) of 16.8 mcg/kg every 4 week-cycle (22). At first dosing (cycle 1, C1), IFNγ increase peaked at around 36 hours, falling near baseline levels around day 8. Diminished peak IFNγ concentrations were observed in most patients at redosing (C2). Middle and lower panels show significant increases in frequencies of activated (Tim3^+^) natural killer (NK) and PD1^+^ NKT cells, and decreased plasmacytoid dendritic cells (pDC) in peripheral blood from 10 patients treated with NHS-IL12 (16.8 mcg/kg) 1 week post cycle 1 versus pre-treatment levels. **(B, C)**. Association between best overall response (BOR) and immune correlates in patients treated with NHS-IL12 in the phase I expansion cohort (55). Immune parameters were compared at baseline to day 14 or day 28 post-treatment in patients treated with 12 mcg/kg (open circles) or 16.8 mcg/kg (closed circles) NHS-IL12 every 2 weeks with a BOR of stable disease (SD) versus progressive disease (PD). Significant differences at baseline between patients with BOR of SD and PD are shown **(B)**. Immune parameters were analyzed at pre- and post-timepoints for patients with BOR of SD and PD, and significant differences in the percent change of immune subsets as a percent of parental cell type from baseline to day 14 and day 28 **(C)** are shown between patients with BOR of SD and PD. Immune subsets with differences between groups are those with p < 0.05 (calculated by the Mann-Whitney test). Bars indicate median with interquartile range. DP, double positive (CD16^+^ CD56^bright^). PBMC, peripheral blood mononuclear cells. **(A)** was adapted from Strauss et al. Clin Cancer Res, 2019. Copyright ^©^ 2019, American Association for Cancer Research. **(B, C)** were adapted from Toney et al., Int Immunopharmacol, 2023. https://doi.org/10.1016/j.intimp.2023.109736; https://creativecommons.org/licenses/by-nc-nd/4.0/. Copyright ^©^ 2023. Published by Elsevier B.V.

In the first-in-human clinical study (NCT01417546) (22), 59 patients with locally advanced or metastatic solid malignancies received NHS-IL12 subcutaneously in single- or multiple-ascending dose cohorts. The primary objective of this study was to identify the maximum tolerated dose (MTD) based on the number of dose-limiting toxicities (DLTs). Twenty-two patients were treated on day 1 and evaluated for 28 days. In the multiple dose-escalation cohorts, 37 patients received NHS-IL12 on day 1, followed by dosing every 4 weeks at 2, 4, 8, 12, 16.8, and 21.8 µg/kg; patients were under observation for at least 6 weeks to evaluate DLTs. NHS-IL12 was well tolerated up to a dose of 16.8 µg/kg (MTD), the recommended dose for phase II studies. Patients enrolled in the multiple ascending-dose cohort received a median of 2.5 doses and were on treatment for a median of 70 days. Patients treated at 16.8 µg/kg received a median of 2 doses (range 1-8). Pharmacodynamic sera analysis at the MTD revealed time-dependent IFNγ elevation after the first dose. Most patients had a less pronounced increase in serum IFNγ after the second dose, which was associated with increased tolerability on the second and subsequent doses (**Figure 4A**). Similar results were observed with IL-10. Toxicity was more pronounced in patients treated at the MTD compared to lower doses. None of the patients treated with single or multiple dosing at up to 12 µg/kg experienced a DLT. One of 6 patients treated at MTD experienced a DLT [grade 3 elevation in alanine transaminase (ALT)]. Two of 6 patients treated above MTD (21.8 µg/kg) experienced a DLT [grade 3 elevations in ALT, aspartate transaminase (AST), and lipase with no signs of pancreatitis]. Of the 59 patients enrolled in the study, grade 3 treatment-related adverse events (TRAEs) were observed in 11 patients, with 1 experiencing a grade 4 TRAE. Decreased lymphocyte count was the most frequent TRAE, observed in 27/59 (45.8%) patients. Additional TRAEs included fever and elevated AST (35.6%), decreased white blood count (WBC; 40.7%), anemia and flu-like symptoms (30.5%), and increased ALT (3.9%). In patients treated at MTD (16.8 µg/kg), elevated AST (75%), increased ALT and decreased WBCs (68.8% each), and decreased lymphocyte count and fever (62.5% each), were the most reported TRAEs. Of 30 evaluable patients, 15 experienced disease stabilization as best overall response, with 5 experiencing durable stable disease (6 to over 30 months). Analysis of PBMCs 1 week after NHS-IL12 dosing revealed increased frequency of activated and mature NK cells, consistent with known IL-12 enhancement of NK cell proliferation and cytotoxicity. NKT cell elevation was also present, lasting up to 2 months post-therapy. Decreases in plasmacytoid DCs (pDC) and terminally differentiated CD4^+^ T cells were also observed. T-cell receptor (TCR) sequencing on matched PBMCs and tumor biopsies from patients with high/medium increase in serum IFNγ demonstrated increased T cell density and TCR diversity at the tumor site, indicative of augmented T cell homing into the tumor. To limit toxicity and determine the optimal treatment schedule and dosing regimen, the study was expanded to include an additional patient cohort treated bi-weekly at 12.0 µg/kg or 16.8 µg/kg (56). Bi-weekly administration was well tolerated, with most adverse events being low grade and self-limiting. Disease stabilization was observed in 50% (6/12) of patients.

Immune activation was more prominent in patients treated with the highest dose, including highest elevations in serum IFNγ, TNFα, and soluble PD-1, and greater increases in frequencies of proliferative and mature NK, CD8^+^T, and NKT cells in peripheral blood. Higher immune activation was also observed in patients treated bi-weekly versus every 4 weeks, namely higher increases in pro-inflammatory serum analytes, proliferative CD8^+^T cells, NK, and NKT cells, intermediate monocytes, and a greater decrease in CD73^+^ T cells. Specific immune analytes at baseline including lower levels of monocytes and plasmacytoid dendritic cells associated with disease stabilization (**Figure 4B**). Analysis of early changes in peripheral blood after NHS-IL12 therapy, including frequency increases in CD8^+^ T cells, immature (CD16^neg^ CD56^bright^) NK cells, and double positive (DP; CD16^+^ CD56^bright^) NK cells were associated with better clinical response (**Figure 4C**).

### 4.2 NHS-IL12 in combination therapy

Based on these clinical findings and promising preclinical data, several clinical studies are currently evaluating NHS-IL12 in combination with various SOC and other immune-oncology agents across tumor types (**Table 1**). A phase I/II clinical study (NCT04633252) is evaluating NHS-IL12 in combination with docetaxel, a tumor necrosis-inducing SOC therapeutic, for the treatment of metastatic prostate cancer. Preliminary data indicate this combination to be safe and with signs of clinical activity (57). In a phase 1b study (NCT02994953), patients with locally advanced or metastatic solid tumors treated with NHS-IL12 and avelumab experienced clinical responses, with some patients achieving prolonged clinical benefit; notably two patients with advanced bladder cancer experienced prolonged complete responses. The safety profile was similar to that observed with individual agents (58).

**Table 1:** Ongoing clinical studies utilizing NHS-IL12 for the treatment of solid malignancies.

Cancer Indication	Combination Approach	Stage	Phase	NHS-IL12 Dose	Trial Identifier
Colorectal, intrahepatic cholangiocarcinoma, bile duct	SOC	Metastatic	Phase II	12 μg/kg, then 8 μg/kg^(1)^	NCT05286814
Cervical, HPV, anal, oropharyngeal, vulvar, vaginal, penile, rectal	Vaccine, M7824	Advanced HPV associated	Phase I/II	16.8 μg/kg, then 8 μg/kg^(2)^	NCT04287868
Oropharyngeal, neck, HPV, anal, cervical, penile, vulvar, vaginal, small bowel, and colon	Entinostat, M7824	Advanced including HPV associated	Phase I/II	4, 8, 12, or 16.8 μg/kg	NCT04708470
Prostate	ADT, SOC, M7824	Metastatic castration sensitive and resistant	Phase I/II	8, 12, or 16.8 μg/kg^(3)^	NCT04633252
	SBRT	Localized high and intermediate risk	Phase II	16.8, 12, or 8 μg/kg^(4)^	NCT05361798
Urothelial, bladder, genitourinary, urogenital	SBRT, M7824	Metastatic non-prostate GU malignancies	Phase I	16.8 μg/kg	NCT04235777

Trials registered at www.clinicaltrials.gov listed with NCT identifiers; access date: 10/03/2023.

ADT, androgen deprivation therapy; HPV, human papillomavirus; M7824, bintrafusp alfa, a bifunctional αPD-L1/TGFβRII fusion protein; SBRT, stereotactic body radiation therapy; SOC, standard-of-care; GU, genitourinary ^(1)^. 12 μg/kg in cycle 1, followed by 8 μg/kg for the remaining cycles ^(2)^; 16.8 μg/kg for the first 4 doses, followed by 8 μg/kg for subsequent dosing; de-escalation to 4 μg/kg allowed ^(3)^. 8, 12, or 16.8 μg/kg in the Phase I cohorts. Phase 2 cohorts dosed at 12 μg/kg ^(4)^. Starting dose 16.8 μg/kg, de-escalated if needed to 12 μg/kg, or 8 μg/kg.

An ongoing phase II trial (NCT04287868) is evaluating NHS-IL12 in combination with bintrafusp alfa (αPD-L1/TGFβRII) and the HPV16 E6/E7 vaccine PDS0101 in patients with HPV-associated malignancies. Clinical activity has been reported for patients with advanced immune checkpoint naïve and refractory HPV16^+^ cancers (59). Objective responses in checkpoint naïve patients were observed in 7/8 patients (88%). Disease reduction was observed in 10/22 (45%) checkpoint-refractory patients, including 6/22 (27%) patients experiencing objective responses. Notably, 5/8 (63%) checkpoint refractory patients receiving 16.8 mcg/kg experienced an objective response versus 1/14 (7%) who received a lower dose (8 mcg/kg), suggesting dose-related response rates, despite similar survival outcomes. Analysis of the peripheral immunome noted combination therapy increased NK cell frequency and pro-inflammatory serum cytokines, while dampening conventional DCs (cDCs) and pDCs, CD4^+^ and CD8^+^ T cells, Tregs, and B cells. Notably, triple therapy increased HPV16-specific T cells > 2-fold in 11/14 patients evaluated. Patients who attained clinical benefit displayed augmented levels of naïve CD8^+^ lymphocytes and decreased cDCs and classical monocytes pre-therapy. An early increase post-therapy in soluble granzyme B, TNFα, and classical monocytes, and a milder decrease in cDCs in peripheral blood also associated with clinical benefit (60).

An ongoing phase II study (NCT04708470) evaluating the combination of NHS-IL12, entinostat, and bintrafusp alfa in advanced colorectal and HPV-associated malignancies has reported preliminary signs of clinical activity in patients with advanced checkpoint refractory HPV16^+^ cancers (61). In this clinical study, 7 patients (5 oropharyngeal, 1 anal, 1 neuroendocrine rectal) were enrolled whose tumors progressed on treatment with the PDS0101 HPV16 vaccine, NHS-IL12 and bintrafusp alfa (NCT04287868). These patients received 300 mg of bintrafusp alfa (i.v.) every 2 weeks, entinostat (p.o.) 3 mg weekly, including a 1 week lead in of entinostat alone, and NHS-IL12 (s.c.) at either 4 mcg/kg every 2 weeks or 8 mcg/kg every 4 weeks (based on dose escalation cohort). Patients receiving NHS-IL12 at 8 mcg/kg did not receive entinostat on the same week but did on all other weeks. Tumor reduction of 28.8%, 38.3% and 42.6% by RECIST was observed in 3/7 (43%) patients. Additional escalating doses of the triple combination of entinostat, NHS-IL12 and bintrafusp alfa continue to be evaluated. Preliminary findings thus indicate the combination of NHS-IL12 with other therapeutics to have a manageable safety profile along with encouraging clinical activity in patients with advanced checkpoint refractory cancers.

## 5 Discussion

The unique biology of IL-12 in bridging innate and adaptive immunity renders this cytokine very promising for agnostic cancer immunotherapy. The pioneering development of NHS-IL12 targeting IL-12 to the tumor site with improved pharmacokinetics and milder IL-12 potency (17, 31, 32) enabled its safe administration to cancer patients (22, 56), overcoming the short half-life and safety challenges of recombinant IL-12 (6, 14, 22). Subcutaneous delivery of NHS-IL12 renders this immunocytokine with an improved safety profile versus traditional intravenous administration of rIL-12, which enables broad systemic cytokine distribution amenable to off-target effects, thereby more prone to the development of immune-related adverse events. In contrast, subcutaneous cytokine delivery allows for lymphatic distribution and target delivery to lymph nodes draining tumor lesions, where T cell priming by dendritic cells is more likely to occur.

Multiple preclinical and clinical studies have demonstrated the tumor suppressive ability of NHS-IL12 as a single agent (22, 35–37, 56). Mounting preclinical and clinical evidence supports the use of NHS-IL12 as an integral component of combination therapies for the treatment of solid cancers, including in combination with immune checkpoint blockade and other treatment modalities (35, 40, 43, 45, 46, 52, 53, 58, 60, 61). A unique aspect of NHS-IL12 versus rIL-12 is its ability to localize to the tumor, thereby eliciting local IFNγ production *via* activation, maturation, and expansion of NK and CD8^+^ TILs. The resulting Th1 responses and heightened cytolytic ability of both NK and CD8^+^ T cells are major drivers of tumor suppression in preclinical studies and in patients (17, 22, 35, 55). This is paralleled with observed decreases in immune suppressive components of the TME, such as TGFβ and infiltrated MDSCs and M2 macrophages, and increased macrophage polarization to an M1 phenotype, which are collectively supportive of tumor control (36, 55).

An important component of the NHS-IL12 mode of action is its ability to modulate antigen presenting cells, including DCs and macrophages. Preclinical studies demonstrated NHS-IL12 to upregulate MHC class I in a dose-dependent manner, suggesting more potent T-cell priming (5, 6, 35). These findings are consistent with decreased circulating plasmacytoid DCs observed in patients treated with NHS-IL12 monotherapy (22, 55). Moreover, the ability of NHS-IL12 to upregulate antigen processing and presentation machinery is consistent with the observed increase in patient antigen-specific T cell responses and TCR diversity (56).

In parallel with the ability to induce pleiotropic tumor-suppressive effects, NHS-IL12 can also drive production of immunosuppressive elements, such as PD-L1, kynurenine, and IL-10 (22, 43, 44, 47, 62). These findings underscore the importance of targeting different components of the TME for an effective cancer therapy involving NHS-IL12. In one example, in a phase 1b study (NCT02994953), patients with metastatic solid tumors treated with NHS-IL12 and avelumab experienced clinical responses, with some patients achieving prolonged partial and complete responses (58).

Tumor-targeted NHS-IL12 is a novel and potent Th1-inducing immunocytokine with the unique ability to engage innate and adaptive immunity, including positive modulatory effects in myeloid populations. The untapped potential of the IL-12 cytokine to enable an effective cooperative engagement of lymphocytes and myeloid cell populations conducive to tumor resolution has been demonstrated in recent preclinical studies with NHS-IL12 in combination with the epigenetic modulator entinostat (52, 53). In these studies, significant antitumor synergy and tumor resolution were mediated through lymphocyte activation and modulation of multiple immunosuppressive myeloid populations to acquire an interferon response able to cooperatively suppress multiple experimental tumor types, including tumors refractory to PD-1/PD-L1-targeted therapies due to defects in MHC I expression, antigen processing and IFNγ signaling. Clinical activity and correlative studies in patients treated in ongoing combination therapy trials involving NHS-IL12 and entinostat may allow a deeper insight into the potential of lymphoid/myeloid crosstalk for cancer therapy.

Clinical responses are being observed in several ongoing clinical studies with NHS-IL12 in combination with one or more agents. Notably, clinical responses are being observed in patients refractory to immune checkpoint blockade who have failed multiple lines of therapy, and progressed in previous combination therapy clinical studies. Future preclinical and clinical studies will inform the most promising indications and combinatorial approaches where NHS-IL12 can bring the most clinical benefit to cancer patients.

## Data availability statement

The original contributions presented in the study are included in the article. Further inquiries can be directed to the corresponding author.

## Author contributions

CM: Writing – original draft, Visualization. GL: Writing – review & editing, Visualization. JG: Writing – review & editing. JS: Conceptualization, Supervision, Writing – original draft, Writing – review & editing. SG: Conceptualization, Supervision, Writing – original draft, Writing – review & editing.
